# Supplementary figures and images for: Sequencing B cell receptors from ferrets (Mustela putorius furo)
Source: PLoS One. 2020 May 29;15(5):e0233794. doi: 10.1371/journal.pone.0233794 (PMC7259655; doi:10.1371/journal.pone.0233794)

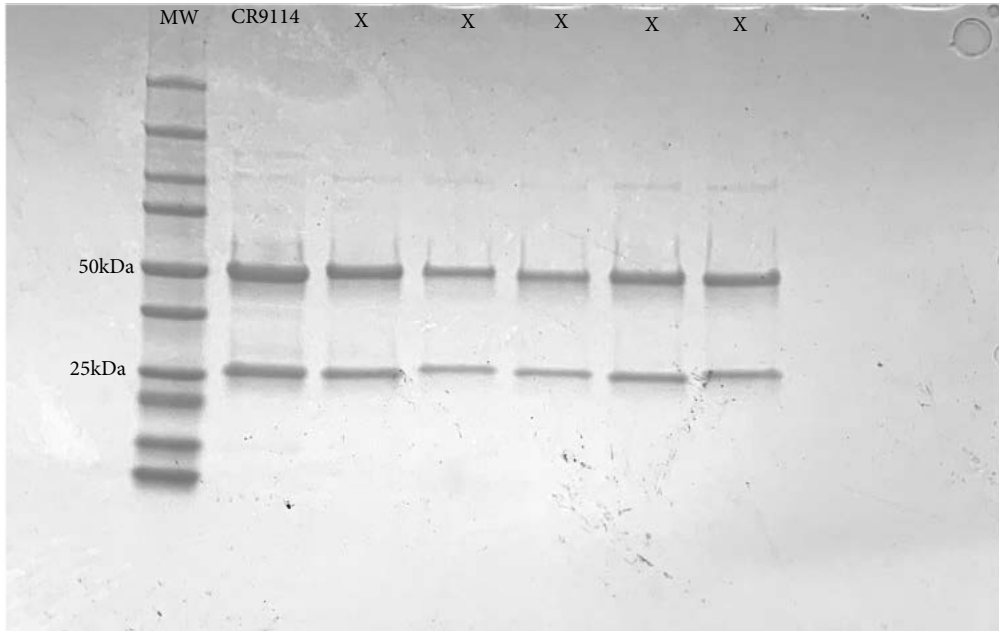

Figure 5A uncropped gel image

Supplement: S1 Raw images — (PDF) [file pone.0233794.s003.pdf]
